# Effect of Synchrotron X-ray Irradiation Time on the Particle Size and DFAFC Performance of Pd/CNT Catalysts

**DOI:** 10.3390/nano14020162

**Published:** 2024-01-11

**Authors:** Sheng-Jung Tsou, Marta Mazurkiewicz-Pawlicka, Yuh-Jing Chiou, Chung-Kwei Lin

**Affiliations:** 1 Department of Chemical Engineering and Biotechnology, Tatung University, Taipei 104-327, Taiwan; eyetoykai@gmail.com; 2 Research Center of Digital Oral Science and Technology, College of Oral Medicine, Taipei Medical University, Taipei 110-301, Taiwan; 3 Faculty of Chemical and Process Engineering, Warsaw University of Technology, 00-645 Warsaw, Poland; marta.pawlicka@pw.edu.pl; 4 School of Dental Technology, College of Oral Medicine, Taipei Medical University, Taipei 110-301, Taiwan

**Keywords:** DFAFC, palladium, formic acid oxidation, synchrotron radiation, photoreduction

## Abstract

Global energy sources are limited, and energy requirements are ever-increasing due to the demand for developments in human life and technology. The environmentally friendly direct formic acid fuel cell (DFAFC) is an attractive electronic device due to its clean energy. In a DFAFC, an anodic catalyst plays an important role concerning the oxidation pathway and activity. In the present study, palladium (Pd) was synthesized by synchrotron X-ray photoreduction using various irradiation times (0.5–4 min) to control the particle size. An acid-treated carbon nanotube (A-CNT) was used as the template for Pd deposition. The A-CNT and Pd/A-CNT were examined using scanning electron microscopy, X-ray diffraction, Raman spectroscopy, and transmission electron microscopy to reveal the microstructural characteristics. Electrochemical evaluation, electrocatalytic activity, and the DFAFC performance of so-obtained Pd/A-CNT catalysts were investigated. The experiment’s results showed that the Pd/A-CNT-2 (i.e., synchrotron photoreduction for 2 min) underwent a direct formic acid oxidation pathway and possessed a high ECSA value of 62.59 m^2^/g_Pd_ and superior electrocatalytic activity of 417.7 mA/mg_Pd_. In a single DFAFC examination, the anodic Pd/A-CNT-2 catalyst had a power density of 106.2 mW/mg_Pd_ and a relatively long lifetime of 2.91 h. Pd/A-CNT-2 anodic catalysts synthesized by surfactant-free synchrotron X-ray photoreduction with a rapid processing time (2 min) are potential candidates for DFAFC applications.

## 1. Introduction

Research and development concerning energy issues are prominent due to limited global resources [1,2,3,4,5,6,7,8,9]. Among these issues of interest are fuel cells, energy conversion devices that can directly convert chemical energy of the fuel with oxidant into water, heat, and electricity [10]. Generally, fuel cells can be divided into various types according to their operation temperature and condition. Abdelkareem et al. [11] classified them into six different types of fuel cells: the alkaline fuel cell (AFC), phosphoric acid fuel cell (PAFC), direct alcohol fuel cell (DAFC), solid oxide fuel cell (SOFC), molten carbonate fuel cell (MCFC), and polymer electrolyte membrane fuel cell (PEMFC). Typical examples concerning individual topics are available in the literature [12,13,14,15,16,17,18]. Among them, the PEMFC is widely applicable, operates easily and is environmentally friendly, and has been regarded as a strategic product by major industrial countries [19,20]. In a PEMFC, the fuel is fed into the anode side and converted into hydrogen ions and electrons. Hydrogen ions then penetrate through the membrane, reduce oxygen at the cathode side, and generate electricity. Fuel cells can continuously supply the electricity within a fuel feed with active catalysts [21].

The polymer electrolyte is a solid membrane that can operate at temperatures up to 90 °C. The voltage for a single PEM fuel cell is 1.1 V and increases with the number of cells connected in a cell stack [22]. PEMFCs accept several types of feeds such as hydrogen, methanol, and formic acid. Formic acid is favored because of its non-toxic and safe features, and this type of PEMFC is called a “direct formic acid fuel cell” (DFAFC). In order to stably supply electricity, the anodic catalyst plays an important role in the lifetime of a DFAFC. When formic acid is fed into the anode side, dual oxidation reaction pathways can occur on the catalysts [23]. The direct oxidation pathway (dehydrogenation reaction) occurs when formic acid is oxidized directly into CO_2_, whereas the indirect oxidation pathway (dehydration reaction) is observed when CO forms as an intermediate during the reaction. CO intermediate tends to adsorb strongly on the surface of the catalyst, which results in poisoning of the catalyst [24]. The CO poisoning reduces the performance and shortens the lifetime of DFAFCs. Thus, this indirect pathway is not preferred and should be avoided.

Not only the lifetime but also the activity of a DFAFC depends on its anodic catalysts. Palladium (Pd) nanocrystals exhibit superior stability and activity. The particle sizes of Pd nanocrystals affect its catalytic ability and stability [25]. For instance, Wang et al. used different concentrations of formic acid to synthesize Pd nanocrystals with various particle sizes. Pd nanocrystals with an average particle size of 4.1 nm exhibited the highest activity of 70 mA/cm^2^ for formic acid oxidation reaction [26]. In addition, Chiou et al. developed a mathematical model to determine the optimal Pd particle size for DFAFC performance and predicted that the cuboctahedron-shaped Pd with a particle size of 2.4 nm is preferred [27].

Pd nanocrystals have been successfully prepared using various methods including NaBH_4_ reduction [28], the polyol process [29], ascorbic acid reduction [30], a hydrothermal method [31], microwave irradiation [32], UV irradiation [33], synchrotron X-ray photoreduction [34], etc. Among the abovementioned technology, synchrotron X-ray photoreduction is a novel preparation method to synthesis noble nanocrystals [35,36,37,38]. Synchrotron radiation is generated by a synchrotron light source for scientific researches [39]. Generally, synchrotron light sources are supported by governments due to their expensive operating cost. Typically, researchers are welcome to use the facility complimentarily, although applications to perform experiments are required beforehand. During synchrotron X-ray irradiation, hydrated electrons (e^−^_aq_) are generated and can be used to reduce the metallic ions. This is a clean, surfactant-free, and instantaneous process for synthesizing nanocrystals [40]. When combined with appropriate templates (such as carbon black, carbon nanotubes, graphene, etc.), the nanocrystals can be homogeneously distributed and enhance the catalytic ability. Among them, the multiwalled carbon nanotube (MWCNT) exhibits unique characteristics of a high specific surface area and conductivity and is a potential candidate to serve as templates for Pd nanocrystal deposition [41,42,43,44]. The raw MWCNT, however, has undesired metallic catalysts and superfluous graphite on the surface. Acid treatment of raw MWCNTs may be mandatory to remove the metallic nanocrystals and generate surface defects for Pd nanocrystal deposition [45,46,47].

In the present work, Pd nanocrystals were synthesized by synchrotron X-ray reduction with various irradiation times. Raw and acid-treated MWCNTs were used as templates for Pd deposition. The so-obtained Pd/CNT nanoparticles were characterized and used as the catalysts for DFAFC application. The effect of synchrotron X-ray irradiation time on the particle size of Pd nanocrystals and further catalytic DFAFC performance was investigated.

## 2. Materials and Methods

### 2.1. Preparation of CNT and Synthesis of Pd-Decorated CNT

Multiwalled carbon nanotubes (MWCNTs; 98~99%, Yong-Zhen Technomaterial Co., Ltd., Taipei, Taiwan) with and without surface treatment were used as templates for palladium deposition. A total of 3 g of commercial MWCNT was immersed in 300 mL of boiling nitride acid (65%, Merck KGaA. Ltd., Darmstadt, Germany) and refluxed for 9 h. After surface treatment, the products were washed with deionized water until the filtrate was neutral (pH = 7). The products were dried at 80 °C for 12 h and used as templates for Pd decoration.

**Figure 1:**
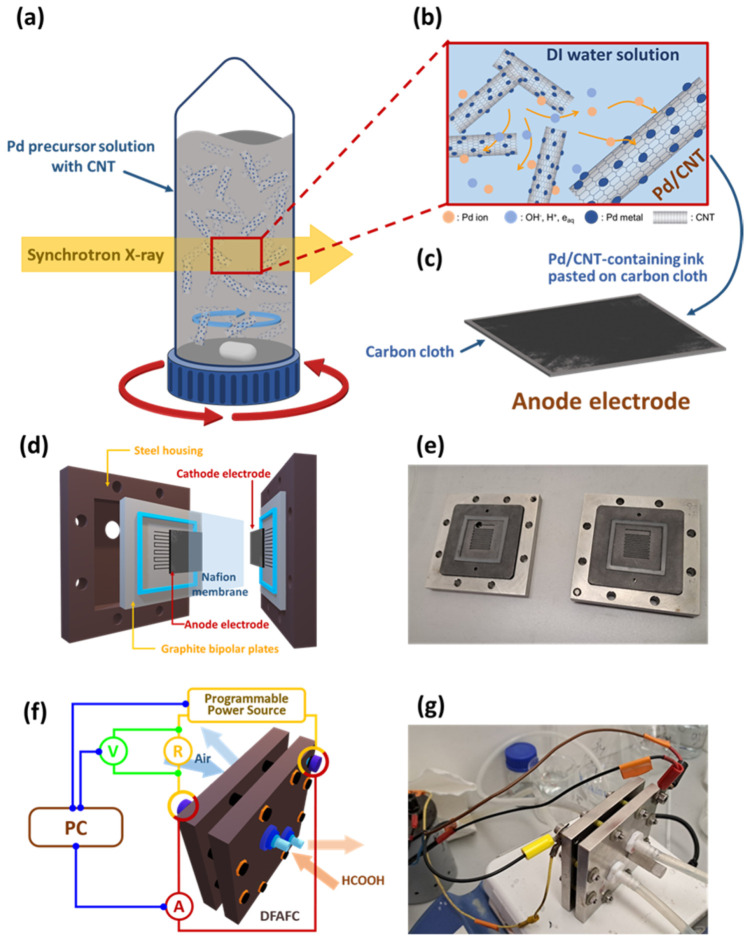
Schematic illustrations of (**a**) synchrotron X-ray irradiation, (**b**) photoreduction of Pd nanocrystals and formation of Pd/CNT, (**c**) anodic catalyst electrode preparation, (**d**,**e**) are schematic diagram and actual photo of DFAFC anode and cathode, (**f**,**g**) are schematic diagram and actual photo of DFAFC single cell assembly for measurement, respectively. The anode and cathode were Pd/CNT and commercially available Pt/C deposited on carbon cloths, respectively. Nafion 115 was used as the membrane.

In total, 20 mg of MWCNT or surface-treated MWCNT (coded as R-CNT and A-CNT, respectively) was added into 40 mL of deionized water and placed in a 50 mL centrifugal tube. The CNT-containing solutions were dispersed ultrasonically for 1 h and then 0.1 mL Pd precursor solution (5 wt.% PdCl_2_ in 10 wt.% HCl) was added. The pH value of the solution was adjusted to 11 by 1 M KOH solution. The precursor-containing centrifugal tube (rotated counterclockwise, notated by red curved arrows) was magnetically stirred (clockwise, blue curved arrows above the magnets within the tube) continuously and exposed to synchrotron X-ray irradiation, as shown in Figure 1a. The synchrotron X-ray was provided by the BL01A beamline of the National Synchrotron Radiation Research Center (NSRRC, Hsinchu, Taiwan). The white-light X-ray beams were trimmed by a pair of slits to obtain a transversal beam section of 13 mm × 9 mm, Figure 1a. Under a stable magnetic stirring, the synchrotron white-light X-ray was irradiated at different times (0.5, 1, 2, and 4 min) to reduce the Pd ion. The so-obtained products (coded as Pd/CNT, as shown in Figure 1b) were washed with DI water and methanol subsequently, then dried at 80 °C for 12 h. The Pd/CNT-containing ink was pasted on carbon cloth to prepare the anode electrode, Figure 1c.

### 2.2. Characterization of R-CNT, A-CNT, and Pd/CNT

The abovementioned materials (R-CNT, A-CNT, and Pd/CNT) were characterized using X-ray diffraction, Raman spectroscopy, field emission scanning electron microscopy, and transmission electron microscopy to reveal their structural and morphological properties. An X-ray diffractometer (XRD, D2 phaser, Bruker, Billerica, MA, USA) operating at 40 KV and 30 mA examined the crystalline structure using nickel-filtered Cu Kα radiation (λ = 0.154 nm). Detailed X-ray analysis was performed by the Rietveld fitting method with XRD analysis software EVA (Bruker-AXS DiffracEVA, Bruker, Billerica, MA, USA) to determine the crystalline size of the synthesized palladium nanocrystals using Scherrer’s formula with a shape factor (k) equal to 0.9 [48,49]. The bonding characteristics of R-CNT and A-CNT were examined by Raman spectroscopy (inVia™ Raman microscope, Renishaw, Wotton-under-Edge, England, UK) to determine the ratio of D-band and G-band. Field emission scanning electron microscopy (FE-SEM, SU 8000 Series UHR, Hitachi, Tokyo, Japan) was used to examine the powder morphology of R-CNT, A-CNT, and Pd/CNT. The detailed crystalline structure and powder morphology were investigated further by using a field emission gun transmission electron microscope (FE-TEM, Tecnai™ G2 F20 S-TWIN, Field Electron and Ion Company, Hillsboro, OR, USA). Brunauer–Emmett–Teller (BET, Porous Materials Inc. BET-201A, Ithaca, NY, USA) measurement was conducted to obtain the specific surface area of the A-CNT and Pd/CNT.

### 2.3. Electrocatalytic Activity of Pd/CNT

The electrochemical performance of the prepared catalysts was evaluated using a three-electrode configuration controlled by a potentiostat (Model 6081C, CH Instrument, Bee Cave, TX, USA). A Pt net was used as the counter electrode, Ag/AgCl saturated with a standard KCl electrode served as a reference electrode, and a catalyst-added SE100-carbon single electrode (Zensor R&D Co., Ltd., Taichung, Taiwan, working diameter 0.5 cm, area 0.196 cm^2^) was used as the working electrode. The catalyst-added working electrode was prepared using the following procedures. Firstly, the catalyst ink was prepared by mixing 1 mg catalyst in 1 mL ethanol and 10 μL 5 wt.% Nafion. The ink solution was sonicated and a calculated volume of catalyst ink was dropped onto the working electrode to produce a uniform catalyst loading of 0.7 mg_cat._/cm^2^. Electrochemical surface area (*ECSA*) and formic acid oxidation (FAO) measurements were examined by using the cyclic voltammetry method. *ECSA* evaluation was performed within −0.2 to 1.5 V (vs. Ag/AgCl) at a scan rate of 50 mV/s using 1 M H_2_SO_4_ as the electrolyte [50]. FAO electrocatalytic activity was determined with the potential ranging from −0.2 to 1.0 V (vs. Ag/AgCl) at a scan rate of 20 mV/s with 3 M HCOOH + 1 M H_2_SO_4_ as the electrolyte [51].

### 2.4. DFAFC Assembly and Evaluation

A direct formic acid fuel cell (DFAFC) has a sandwich structure consisting of two graphite bipolar plates, two electrodes (anode and cathode), a Nafion proton exchange membrane, and a steel housing. The anode electrode used in the experiments underwent a sequence of preparation procedures. Firstly, 25 mg of Pd/CNT catalyst (corresponding to 0.4 mg/cm^2^ of Pd loading) was dispersed in ultrapure water. A total of 5 wt.% isopropanol-based Nafion dispersion was added into the as-prepared solution to obtain the desired anodic catalyst ink. The anode electrode was prepared by pasting the anodic ink in an area of 2.6 × 2.6 cm^2^ of Carbon Cloth CC6 Plain (Fuel Cell Store, Bryan, TX, USA). The cathode was prepared through similar procedures, except a different catalyst and carbon cloth were used. Commercially available 40 wt.% Pt/Vulcan XC-72 (Premetek, Cherry Hill, NJ, USA) was used as the cathodic catalyst (corresponding to 3 mg/cm^2^ of Pt loading) that was pasted on the surface of the Carbon Cloth CC4 Wet Proofed (Fuel Cell Store, Bryan, TX, USA). The prepared electrodes were dried at 130 °C under an applied pressure of 0.3 MPa. Simultaneously, the Nafion^TM^ 115 (Fuel Cell Store, Bryan, TX, USA) membrane was conditioned using 5% H_2_O_2_ (Chempur, Piekary Śląskie, Poland), 0.5 M H_2_SO_4_ (Chempur, Piekary Śląskie, Poland), and ultrapure water. Figure 1d,e show the schematic illustration and the actual photo of DFAFC components, respectively.

DFAFC performance was investigated by using a single cell with a home-made evaluation system [52]. The components as shown in Figure 1e were assembled into the steel house and connected to the evaluation system. DFAFC activity and stability tests were investigated by using a 3 M formic acid solution at a flow rate of 3 mL/min and air in a flow rate of 1240 sccm for anode and cathode fuel, respectively. The polarization curves were obtained by scanning the voltage response of the DFAFC across the current ranging from 0 to 400 mA/g_Pd_. The stability tests were carried out at a constant current that was determined by 80% of the highest power density. During the tests, the DFAFC was maintained at a constant temperature of 40 °C. Figure 1f,g show the schematic illustration and the actual photo of the DFAFC evaluation system, respectively.

## 3. Results and Discussion

### 3.1. Characterization of R-CNT and A-CNT

**Figure 2:**
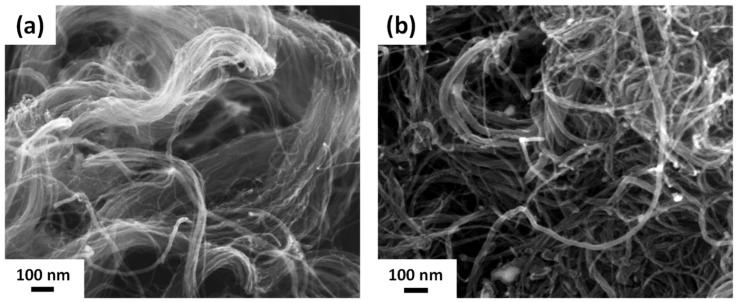
FE-SEM images of (**a**) R-CNT and (**b**) A-CNT.

Multiwalled carbon nanotubes (CNTs) served as templates for Pd deposition. Two types of CNTs (raw and acid-treated, denoted as R-CNT and A-CNT, respectively) were used in the present work. Figure 2 shows the SEM images before (R-CNT) and after acid treatment (A-CNT). It can be noted that R-CNT, Figure 2a, exhibited a bundle-like structure with superfluous graphite. After acid treatment (Figure 2b), the superfluous graphite was removed and the surface of A-CNT was relatively smooth compared to that of R-CNT as shown in Figure 2a.

**Figure 3:**
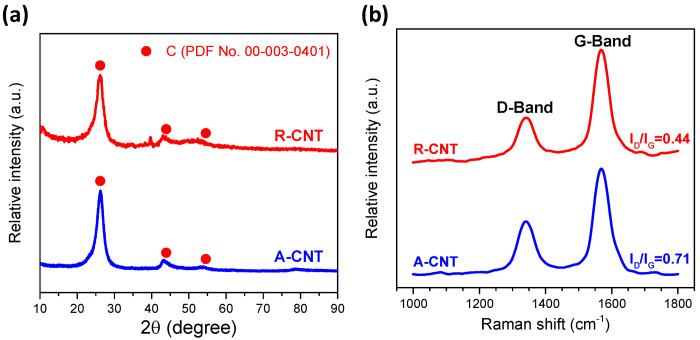
(**a**) X-ray diffraction patterns and (**b**) Raman spectra of R-CNT and A-CNT.

X-ray diffraction was used to characterize the crystalline structure of R-CNT and A-CNT and Figure 3a shows the corresponding XRD patterns. Before acid treatment, R-CNT (upper red curve), exhibited a typical carbon graphite structure (PDF No. 00-003-0401) with a relatively large background and some minor diffraction peaks (probably due to the catalysts used for synthesis of multiwalled carbon nanotubes). After acid treatment, A-CNT (bottom blue curve), showed a distinct carbon graphite structure without minor phases. The direct bonding of CNT (D-band and G-band corresponding to diamond sp^3^ and graphite sp^2^ bonding, respectively) was examined by Raman spectroscopy and Figure 3b shows the corresponding Raman spectra of R-CNT and A-CNT. Both R-CNT and A-CNT exhibited distinct D-band and G-band peaks at 1340 cm^−1^ and 1566 cm^−1^, respectively, and show similar results to those reported in the literature [53]. The ratio between D-band and G-band, however, can be used as an indicator of disorder and defects in R-CNT and A-CNT [54,55], and the ratios were 0.44 and 0.71 for R-CNT and A-CNT, respectively. After acid treatment, the catalysts used for CNT preparation and superfluous graphite (containing sp^2^ only) were removed. Thus, the significant increase in the I_D_/I_G_ ratio indicated a large increase in defects for A-CNT, which can be beneficial for Pd deposition.

### 3.2. Characterization of Pd/CNT

Although both R-CNT and A-CNT were used as the templates for Pd deposition, the desired Pd-decorated R-CNT (i.e., Pd/R-CNT) was not prepared successfully. Though not shown here, the as-synthesized products were examined using SEM and X-ray diffraction. X-ray diffraction patterns showed a mixture of both carbon graphite and Pd phases. The SEM images of as-prepared Pd/R-CNT, however, were similar to those of R-CNT, as shown in Figure 2a. This suggested that the as-prepared products were a mixture of Pd nanocrystals and R-CNT. Limited desired Pd/R-CNT was observed. This may be attributed to the insufficiency of defects in R-CNT, which resulted in a lack of required nucleation sites for Pd deposition. This shows a similar trend as that reported in the literature [56] where Kim et al. suggested that the defects acted as nucleation sites for the deposition of metallic nanocrystals. Further investigations focused on Pd/A-CNTs.

**Figure 4:**
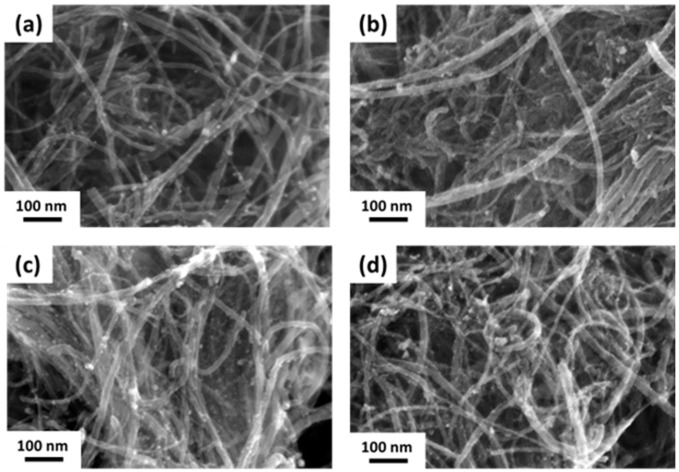
FE-SEM images of Pd/A-CNT after synchrotron X-ray irradiation for (**a**) 0.5, (**b**) 1, (**c**) 2, and (**d**) 4 min, respectively.

**Figure 5:**
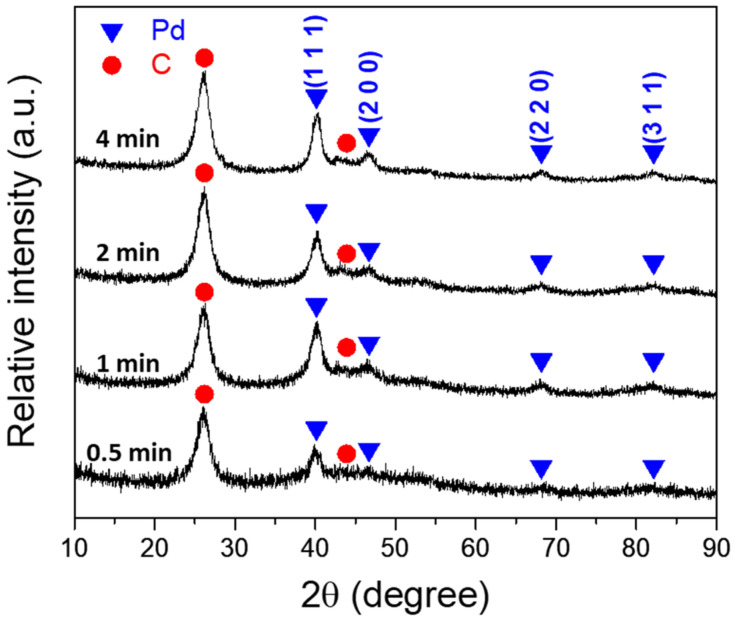
X-ray diffraction patterns of Pd/A-CNT as a function of synchrotron X-ray irradiation time.

Figure 4 shows a series of SEM images of Pd/A-CNT synthesized by the synchrotron X-ray photoreduction method [57,58,59]. It can be noted that Pd nanocrystals were deposited successfully onto A-CNT, shown as bright dots in Figure 4. At a relatively short irradiation time (0.5 min, Figure 4a), only a small amount of Pd nanocrystals were deposited on A-CNT. With increasing irradiation time, more Pd/A-CNT was observed. However, no significant differences were observed after synchrotron X-ray photoreduction for 1–4 min, Figure 4b–d. X-ray diffraction was further used to confirm the formation of Pd nanocrystals, and Figure 5 shows the corresponding XRD patterns of Pd/A-CNT after irradiation for 0.5–4 min, respectively. A shown in the bottom XRD curve, after 0.5 min of photoreduction, relatively small Pd diffraction peaks (PDF No. 03-065-6174) at 40.16°, 46.35°, 49.85°, and 59.11° from the (111), (200), (220), and (311) crystalline planes, respectively, were observed. With increasing irradiation time, the intensity of these Pd diffraction peaks increased. This indicated a relatively small amount of Pd nanocrystals formed at the beginning of irradiation and the amount of Pd nanocrystals increased with increasing irradiation time. This confirmed the observation seen using SEM in Figure 4.

**Figure 6:**
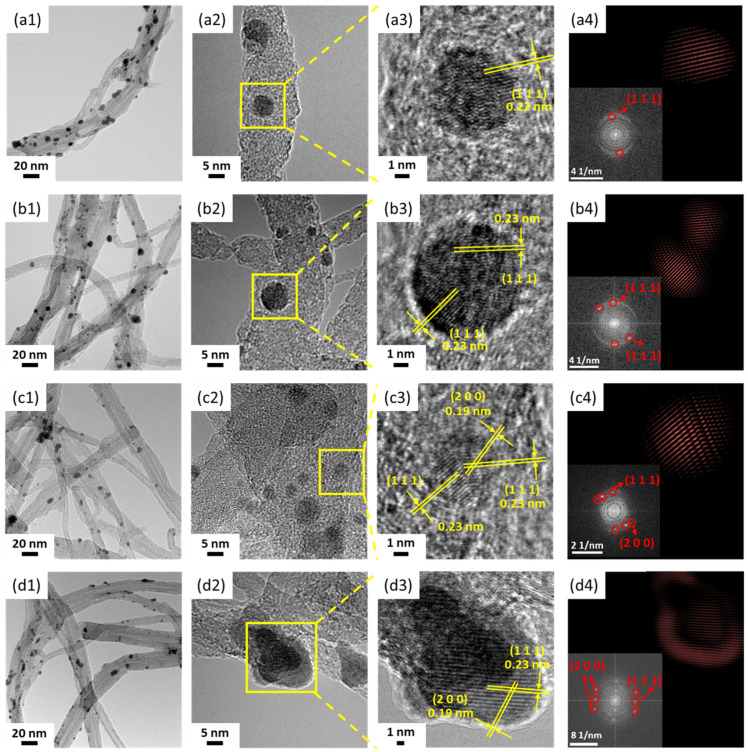
TEM images, fast Fourier-transform (FFT), and inverse FFT patterns of Pd/A-CNT after synchrotron X-ray irradiation times of 0.5 min (**a1**–**a4**), 1 min (**b1**–**b4**), 2 min (**c1**–**c4**), and 4 min (**d1**–**d4**). The FFT patterns are shown as the inserted figures in (**a4**–**d4**).

Transmission electron microscopy (TEM) was performed to better reveal the morphological and crystalline structure of the as-prepared Pd/A-CNT nanocrystals, Figure 6. After 0.5 min of synchrotron X-ray irradiation, Figure 6(a1) shows that Pd nanocrystals were deposited homogeneously on A-CNT. At a higher magnification (Figure 6(a2)), it was noted that the spherical Pd nanocrystals were embedded on the defects of A-CNT. Figure 6(a3) shows one of these Pd nanocrystals at a higher magnification where layered Pd atoms were better observed. The image of Figure 6(a3) was examined further using Image J software, and the fast Fourier-transformation (FFT) pattern (the bottom inserted figure in Figure 6(a4)) and the inverse FFT pattern (Figure 6(a4)) were obtained. The (111) crystalline plane of Pd nanocrystal with a d-spacing of 0.22 nm can be clearly determined. Similar trends were observed for Pd/A-CNT with a relatively longer irradiation time (1–4 min, Figure 6(b1–d4)). However, it should be pointed out that with a longer synchrotron X-ray irradiation time (>2 min), the d-spacing of the (111) plane slightly increased to 0.23 nm and an extra crystalline plane of (200) with a d-spacing of 0.19 nm can be observed, Figure 6(c4,d4). This shows results similar to those seen in the X-ray diffraction patterns, Figure 5.

**Figure 7:**
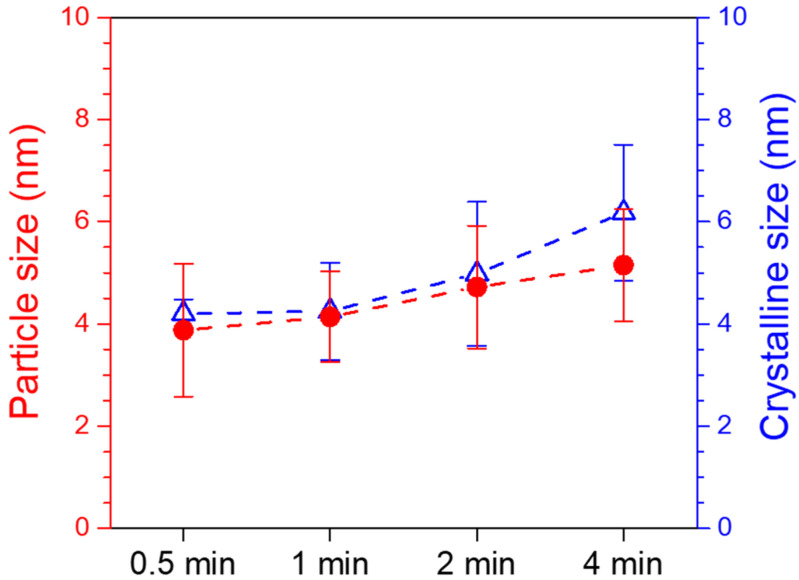
The particle size and crystalline size of Pd nanocrystals measured from TEM images and calculated by X-ray diffraction patterns, respectively.

The particle size of the Pd nanocrystals was measured by examining the TEM images (as shown in Figure 6, at least 100 nanocrystals from several photos were examined), and Figure 7 (red solid circle symbols) shows the corresponding results where the particle sizes were 3.9 ± 1.3, 4.1 ± 0.9, 4.7 ± 1.2, and 5.2 ± 1.1 nm after photoreduction for 0.5, 1, 2, and 4 min, respectively. The crystalline size was calculated by Scherrer’s formula [60] and the estimated crystalline sizes (blue hollow triangle symbols) were slightly larger than those of the particle sizes (from TEM measurement), and they were 4.2 ± 0.1, 4.3 ± 1.0, 5.0 ± 1.4, and 6.2 ± 1.3 nm, respectively. Both the particle sizes and the crystalline sizes of the Pd nanocrystals were very close without any statistical differences. They did, however, show a similar trend, and they increased with increasing irradiation time.

### 3.3. Electrocatalytic Activity and DFAFC Performance

As demonstrated above, Pd/A-CNT consisted of Pd nanocrystals with sizes ranging from 3.9 to 5.2 nm (by TEM) and embedded in the defects of acid-treated carbon nanotubes (A-CNT). The slight differences in particle size (or crystalline size) due to the synchrotron irradiation time may result in differences in electrocatalytic activity and DFAFC performance.

The performance of electrocatalytic activity may be affected by the surface active sites that may be revealed by the specific surface area and electrochemical active surface area (i.e., *ECSA*). The specific surface area was determined by the BET method, and these were 233.48 and 169.38 m^2^/g for A-CNT and Pd/A-CNT-2, respectively. A significant decrease in specific surface area was observed after synchrotron X-ray reduction of Pd nanocrystals. It should be pointed out that A-CNT possessed a high specific surface area (233.48 m^2^/g) that served as the nucleation site for Pd reduction. Thus, a relatively small specific surface area for Pd/A-CNT-2 was noticed. It should be pointed out that BET examinations revealed the specific surface area by nitrogen absorption (that was mainly contributed by A-CNT) and *ECSA* was determined by Pd catalysts. Further investigations focused on *ECSA*.

**Figure 8:**
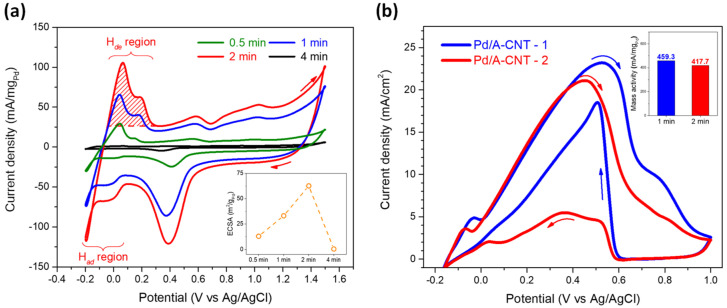
(**a**) Cyclic voltammetric curves of various Pd/A-CNTs. The insert in the figure shows the calculated *ECSA* by integrating each hydrogen desorption (H*_de_*) area. The test was performed in 1 M H_2_SO_4_ electrolyte at a scan rate of 50 mV/s. (**b**) Cyclic voltammetric curves for formic acid oxidation activity evaluation. The test was performed in 3 M HCOOH + 1 M H_2_SO_4_ electrolyte at a scan rate of 20 mV/s for a better resolution of the reaction.

Figure 8a shows the cyclic voltammetry measurement results in the 1 M H_2_SO_4_ electrolyte at a scan rate of 50 mV/s. As a general trend, within the anodic scan, hydrogen desorption, a double layer, Pd oxidation, and oxygen generation were noticed subsequently [61,62]. The hydrogen desorption from active sites of the Pd catalyst (H*_de_*) was observed within −0.2 V to 0.3 V. After hydrogen desorption, a double layer formed due to water molecular reversion within 0.3 to 0.7 V. With a further increase in potential (0.7–1.1 V), oxidation of the Pd catalyst occurred. Finally, oxygen generation was observed with a potential higher than 1.1 V. A cathodic scan, reduction of Pd, reverse double layer, and hydrogen absorption may occur [63,64]. It should be pointed out that the electrochemical activity differed for Pd/A-CNT with different photoreduction times. The electrochemical performance increased gradually with increasing photoreduction time and reached a maximum for the Pd/A-CNT-2 catalyst. A drastic decrease in electrochemical performance, however, was noticed for the Pd/A-CNT-4 catalyst. This indicated that a small increase in particle size (or crystalline size) may result in a significant decrease in the surface active site of the Pd catalyst. With the obtained CV curves, the electrochemical active surface area (*ECSA,* m^2^/g_Pd_) of the Pd/A-CNT catalyst was calculated according to the following equation [65,66,67]:

$$ECSA=\frac{Q_{H_{de}}}{W_{Pd}\cdot Q_{ref}}\;$$

where *Q_Hde_* (C/m^2^) is the charge of H*_de_* that is calculated by integrating the CV curve in the H*_de_* region (for instance, the red back slash of Pd/A-CNT-2 in Figure 8a). *W_Pd_* (g_Pd_/m^2^) is the specific Pd loading amount and *Q_ref_* is the amount of H*_de_* per unit of catalyst surface area (for Pd, *Q_ref_* = 4.1 C/m^2^) [62,63].

As shown in the bottom right insert figure of Figure 8a, the *ECSA*s were 13.02, 33.14, 62.59, and 0.41 m^2^/g_Pd_ for photoreduction times of 0.5, 1, 2, and 4 min, respectively. They were better than those reported in the literature. For instance, Ma et al. used the commercial Pd/C and used oleylamine and octadecylene as reducing agents to synthesize Pd/C for formic acid oxidation [68] and the *ECSA*s were 13.3 and 18.0 m^2^/g_Pd_, respectively. Chen et al. [69] synthesized boron-doped graphene and decorated with a Pd nanocrystalline catalyst, and the resulting *ECSA* was 46.20 m^2^/g_Pd_. Pd/A-CNT-1 and Pd/A-CNT-2 exhibited superior *ECSA* values (33.14 and 62.59 m^2^/g_Pd_, respectively) and were examined further concerning their formic acid oxidation activities.

Figure 8b shows the electrocatalytic performance of the Pd/CNT-1 and Pd/CNT-2 catalysts where the formic acid oxidation activity was examined in a 3 M HCOOH + 1 M H_2_SO_4_ mixed electrolyte at a scan rate of 20 mV/s within the potential range of −0.2 V to 1.0 V. It should be pointed out that the scan rate for electrocatalytic performance evaluation was set at a relatively low speed of 20 mV/s to have a better resolution of the formic acid oxidation reaction. As shown by the blue curve in Figure 8b, Pd/A-CNT-1 exhibited a small peak at −0.03 V, followed by a large oxidation peak at 0.53 V with a maximum current density of 23.2 mA/cm^2^. A shoulder at 0.78 V was observed before the end of the anodic scan. For a formic acid oxidation reaction, there are direct and indirect pathways during the anodic scan [70]. The direct pathway is the dehydrogenation reaction within 0.0–0.5 V in which the formic acid is directly oxidized to CO_2_ and a hydrogen ion (H^+^). In the indirect pathway (0.5–0.9 V), the formic acid was firstly oxidized to CO, H_2_O, and a hydrogen ion (H^+^). Subsequently, the CO intermediate was adsorbed on the catalyst surface and oxidized further to CO_2_. The indirect pathway is not preferred due to its possible poisoning of catalysts [71].

The first small peak at −0.03 V was attributed to the hydrogen desorption [72]. The actual oxidation of formic acid by the Pd/A-CNT-1 catalyst that occurred at 0.53 V was probably due to a combination of direct and indirect oxidation pathways, whereas the shoulder peak at 0.78 V was definitely from the indirect formic acid oxidation reaction. For Pd/A-CNT-2 (the red curve in Figure 8b), distinct direct oxidation of formic acid at 0.45 V was observed. In addition, the indirect-pathway shoulder was almost absent. This indicated that almost direct oxidation occurred with the assistance of the Pd/A-CNT-2 electrocatalyst.

The maximum current densities were 23.2 and 21.1 mA/cm^2^ for Pd/A-CNT-1 and Pd/A-CNT-2, respectively. After normalizing with its loaded weight of Pd nanocrystals, the corresponding mass activities were derived, and they were 459.3 and 417.7 mA/mg_Pd_ for Pd/A-CNT-1 and Pd/A-CNT-2, respectively. Though Pd/A-CNT-1 exhibited a higher mass activity than that of Pd/A-CNT-2, it should be pointed out that the formic acid oxidation pathway was different. The high current density of Pd/A-CNT-1 (23.2 mA/cm^2^) compared to that of Pd/A-CNT-2 (21.1 mA/cm^2^) was attributed to the indirect formic acid oxidation. Pd/A-CNT-2 exhibited direct formic acid oxidation and is expected to have better electrocatalytic performance than that of Pd/A-CNT-1, for which an indirect oxidation reaction occurred.

**Figure 9:**
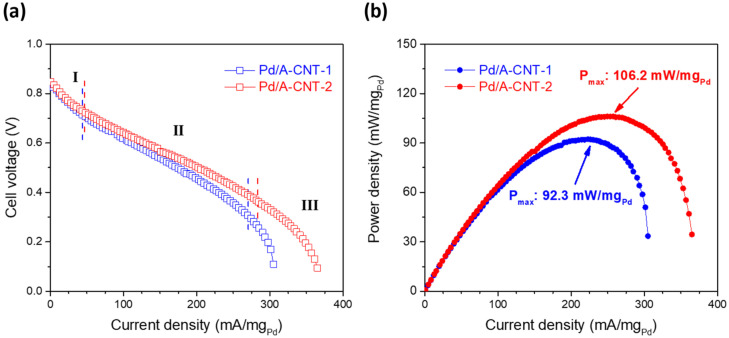
(**a**) Polarization curves and (**b**) power density of DFAFC where the test was performed in 3 M HCOOH with a flow rate of 3 mL/min.

The performance of the direct formic acid fuel cell was evaluated based on its activity and stability. Figure 9a shows the polarization curve that was obtained by applying the current density (0–400 mA/mg_Pd_) and recording the corresponding voltage response of a single fuel cell. Figure 9b shows the cell voltage and power density of the direct formic acid fuel cell with the Pd/A-CNT-1 and Pd/A-CNT-2 catalysts. As shown in Figure 9a for the Pd/A-CNT-1 catalyst (blue hollow square symbols), the open voltage was 0.826 V and the activity polarization loss was observed at a current density ranging from 0 to 40 mA/mg_Pd_. A linear current density was noticed within an applied current density ranging from 40 to 270 mA/mg_Pd_ due to ohmic polarization loss. Another rapid concentration loss was observed and the final current density reached 304.92 mA/mg_Pd_. Though similar behavior was observed, the performance was improved by using Pd/A-CNT-2 as the catalyst (red hollow square symbols). The open circuit voltage was 0.847 V, which was similar to that of Pd/A-CNT-1. The activity, ohmic, and concentration polarization losses were shifted to higher current densities, and these were 0.0–48.2, 48.2–284.6, and 284.6–364.9 mA/mg_Pd_, respectively. Figure 9b shows the corresponding power density (calculated by multiplying the applied current density with cell voltage), which reveals the performance of the formic acid fuel cell more clearly. The maximum power density was 106.2 mW/mg_Pd_ at a current density of 249.4 mA/mg_Pd_ for Pd/A-CNT-2, whereas it was 92.3 mW/mg_Pd_ at a current density of 222.8 mA/mg_Pd_ for Pd/A-CNT-1. It is apparent that Pd/A-CNT-2 exhibited a superior anodic catalytic activity than that of Pd/A-CNT-1. The catalytic performance of Pd/A-CNT investigated in the present study was compared with those reported in the literature and summarized in Table 1.

**Figure 10:**
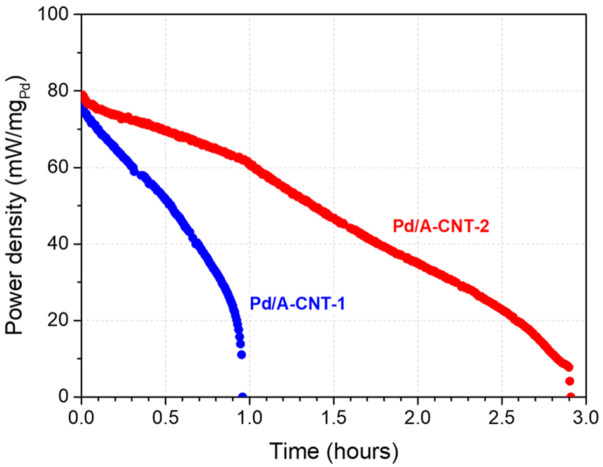
The stability measurement of DFAFC where the test was performed in 3 M HCOOH with a flow rate of 3 mL/min.

Figure 10 shows the stability evaluation of the direct formic acid fuel cell with Pd/A-CNT as the catalyst. For Pd/A-CNT-1 (blue solid circle symbols), a small decrease in power density from 76.34 to 74.26 mW/mg_Pd_ was observed at the beginning of the examination. This was probably due to the accumulation of CO_2_ gas bubbles on the anode electrode layer [79]. After the small drop, the power density decreased continuously and deactivated at 0.96 h. Pd/A-CNT-2 (red solid circle symbols), however, exhibited superior performance compared to that of Pd/A-CNT-1. The initial power density was 79.03 mW/mg_Pd_ and dropped to 76.72 mW/mg_Pd_. Thereafter, a continuous decrease in power density from 76.72 mW/mg_Pd_ was observed and deactivated at 2.91 h. It is obvious that Pd/A-CNT-2 exhibited a longer lifetime compared to that of Pd/A-CNT-1. Recalling Figure 9b, Pd/A-CNT-1 and Pd/A-CNT-2 followed different formic acid oxidation reaction pathways. Pd/A-CNT-1 exhibited an indirect oxidation pathway. The active sites of the catalyst were easily poisoned and catalytic activity decreased quickly. This resulted in a relatively short lifetime for Pd/A-CNT-1. In contrast, the direct formic acid fuel cell with Pd/A-CNT-2 catalyst exhibited a direct oxidation pathway, better stability, and longer lifetime.

Compared to the initial power density, Pd/A-CNT-2 exhibited relative power densities of 77, 44, and 10% at 1 h, 2 h, and 2.9 h, respectively. Ha et al. [80] and Larsen et al. [74] examined the performance of a commercial Pd catalyst and reported ~62% and 63%, respectively, of the initial power density after 0.95 h of testing. The catalytic performance of the commercial Pd and MWCNT powder mixture was examined by Chang et al. and exhibited a relative power density of 55% after 2 h of examination [76]. Zhu et al. [75] found that Pd catalytic performance was affected by their fuel concentration and testing temperature. Excellent stability and a longer lifetime (44% after 6.2 h evaluation) were revealed when tested at a concentration of 10 M formic acid and 20 °C. The DFAFC durability performance of Pd/A-CNT investigated in the present study was compared with those reported in the literature and summarized in Table 2. Though the evaluation parameters for a single cell of a DFAFC were different for the various researches, the present Pd/A-CNT-2 catalysts synthesized by synchrotron X-ray photoreduction can be optimized further for practical DFAFC applications.

## 4. Conclusions

In the present study, acid-treated carbon nanotubes (A-CNT) were used as a template and decorated with synchrotron-photoreduced Pd nanocrystals. The particle size of Pd nanocrystals increased from 3.9 to 5.2 nm with increasing irradiation time for 0.5–4 min, respectively. Pd/A-CNT powders were used as electrocatalysts for formic acid oxidation and Pd/A-CNT-1 and Pd/A-CNT-2 (i.e., X-ray photoreduction for 1 and 2 min, respectively) exhibited superior ECSAs of 33.14 and 62.59 C/m^2^, respectively. Pd/A-CNT-1 exhibited an indirect oxidation pathway, whereas Pd/A-CNT-2 followed a direct oxidation pathway. Pd/A-CNT-2, possessing a mass activity of 417.7 mA/mg_Pd_, superior power density of 106.2 mW/mg_Pd_, and extended lifetime of 2.91 h, has potential to be used as a novel anodic catalyst for direct formic acid fuel cell applications.

## Figures and Tables

**Table 1:** Comparison of DFAFC activity performances according to different literature.

Sample (Anode)	Preparation	Cathode	Max. Power Density (mW/mg_Pd_)	Ref.
Pd	Electrodeposit	Pt	7.5	[73]
Pd Black	NaBH_4_ reduction	Pt nanoparticle	108.3	[74]
Pd Black	Commercial	Pt nanoparticle	31.6	[75]
Pd polyhedron	HCOOH reduction	Pt GDE	202.0	[53]
Pd/C	Commercial Pd and C mixture	Pt nanoparticle	81.8	[76]
Pd/C	Commercial	40 wt.% Pt/C	22.9	[77]
Pd/MWCNT	X-ray photoreduction	60 wt.% Pt/C	241.0	[27]
Pd/*f*-MWCNT	Polyol method	60 wt.% Pt/C	175.8	[27]
Pd/purified MWCNT	NaBH_4_	60 wt.% Pt/C	199.4	[27]
Pd/*f*-CNT	High-pressure microwave	60 wt.% Pt/C	75.0	[78]
Pd/*f*-CNT	Microwave-assisted polyol	60 wt.% Pt/C	57.0	[78]
Pd/*f*-CNT	NaBH_4_ reduction	60 wt.% Pt/C	119.0	[78]
Pd/A-CNT-1	X-ray photoreduction	40 wt.% Pt/C	92.3	This work
Pd/A-CNT-2	X-ray photoreduction	40 wt.% Pt/C	106.2	This work

**Table 2:** Comparison of DFAFC durability performance according to different literature.

Sample (Anode)	Formic Acid Concentration	Lifetime (Hour)	Percentage of Initial Power Density	Ref.
Commercial Pd	5 M	0.95	62%	[80]
Commercial Pd	5 M	0.95	63%	[74]
Pd/C	3 M	2.00	55%	[76]
Pd Black	10 M	6.20	44%	[75]
Pd/CNT-2	3 M	2.90	10%	This work *

* After 1 h and 2 h of testing, the retained percentages of initial powder density were 77 and 44%, respectively.

## Data Availability

The data presented in this study are available on request from the corresponding author.
